# The Health-Promoting Quality Attributes, Polyphenols, Iridoids and Antioxidant Activity during the Development and Ripening of Cornelian Cherry (*Cornus mas* L.)

**DOI:** 10.3390/antiox13020229

**Published:** 2024-02-13

**Authors:** Dominika Przybylska, Alicja Z. Kucharska, Narcyz Piórecki, Tomasz Sozański

**Affiliations:** 1 Department of Fruit, Vegetable and Plant Nutraceutical Technology, Wrocław University of Environmental and Life Sciences, Chełmońskiego 37, 51-630 Wrocław, Poland; 2 Arboretum and Institute of Physiography in Bolestraszyce, 37-700 Przemyśl, Poland; npiorecki@ur.edu.pl; 3 Institute of Physical Culture Sciences, Medical College, University of Rzeszów, Cicha 2A, 35-326 Rzeszów, Poland; 4 Department of Preclinical Sciences, Pharmacology and Medical Diagnostics, Faculty of Medicine, Wroclaw University of Science and Technology, Wybrzeże Wyspiańskiego 27, 50-370 Wrocław, Poland; tomasz.sozanski@pwr.edu.pl

**Keywords:** *Cornus mas* L., fruit ripening, physicochemical properties, flavonoids, phenolic acids, ellagitannins, gallotannins, iridoids

## Abstract

This study defined the physicochemical attributes, composition, and antioxidant capacity of four Polish cultivars of cornelian cherry (CC) at six stages of development and ripening. A total of 52 metabolites were identified by UPLC-ESI-qTOF-MS/MS and quantified by HPLC-PDA. In general, phenolic acids, hydrolyzable tannins, flavonols, iridoids, antioxidant activity, organic acids, and vitamin C decreased, while anthocyanins, malic acid, sugars, and titratable acidity increased. For the first time, we determined the evolution of the CC chemical properties and the metabolic behavior and quantified the individual compounds, and groups of compounds during ripening, in particular gallotannins, ellagitannins, iridoids, and organic acids. The main novelty of our study is that CC is a valuable resource for utilization at different degrees of maturity. We showed that unripe fruits in particular deserve valorization, as they contained the highest content of total bioactive phytocompounds (5589.1–6779.6 mg/100 g dw)—primarily phenolic acids > iridoids > tannins—and the highest antioxidant capacity. The intermediate stages were the most abundant in vitamin C (341.1–495.6 mg/100 g dw), ellagic acid (5.9–31.6 mg/100 g dw), gallotannins (47.8–331.1 mg/100 g dw), and loganic acid (1393.0–2839.4 mg/100 g dw). The ripe fruits contained less bioactive phytocompounds (1403.7–1974.6 mg/100 g dw)—primarily iridoids > phenolic acids > tannins > anthocyanins—and the lowest antioxidant capacity. On the other hand, ripe fruits showed the highest content of anthocyanins (30.8–143.2 mg/100 g dw), sugars (36.4–78.9 g/100 g dw), malic acid (5.5–12.2 g/100 g dw), and, favorably for the nutritional applications, the highest sugar-to-acids ratio (3.0–6.4). Our work illustrates in detail that quality attributes and the content of health-promoting phytocompounds in CC depend on the ripening stage and on the cultivar. These results advance the scientific knowledge about CC. Our findings can be helpful to select the optimal properties of CC for the development of diverse functional foods and phytopharmaceuticals applied in the prevention of civilization diseases.

## 1. Introduction

The increased awareness of the diet-to-health relation has led to multiple studies focusing on fruits rich in bioactive compounds with health-promoting potential. One such fruit is the cornelian cherry, CC (*Cornus mas* L.), which has long been utilized in traditional curative practices and for nutritional applications. CCs contain simple sugars (glucose, fructose, and trace amounts of sucrose), proteins, organic acids, pectin, lipids, vitamins C, A, E, B2, B7, and certain elements—K, Na, Ca, Mg, P, Fe, Zn, Cu, and Mn [1,2].

Ripe CC has been widely discussed in the scientific literature over the last decades. The fruits of this plant, as well as its flowers, leaves, and stones, provide an array of chemical compounds, including natural antioxidants [1,3,4,5,6]. They contain polyphenols (phenolic acids, hydrolysable tannins, anthocyanins, flavonols), iridoids, vitamin C, fiber, and unsaturated fatty acids [1,2]. The consumption of such fruits, rich in natural antioxidants, can reduce the alterations triggered by reactive oxygen species and increase the antioxidant defense of the human organism against the development of various diseases. As of today, research has confirmed many health-promoting effects of both individual compounds present in CC or their synergistic action, e.g., antioxidative (polyphenols) [5,7], anti-inflammatory (loganic acid) [8], antidiabetic (homogenized fruits, anthocyanins gallotannins and ellagitanins) [9,10,11], anti-osteoporotic (loganic acid) [12], antibacterial (tellimagrandin I) [13,14], hepatoprotective (anthocyanins, iridoids, ellagitannins, CC extract) [15,16,17], antiglaucomic (loganic acid) [18], anti-lipidemic, anti-atherosclerotic (dried fruits, fruit extract, loganic acid, anthocyanins) [8,19,20,21], anticancer (ellagitannins) [22], and other effects of ripe CC [1,8,23]. Improper diet and insufficient physical activity are two major factors contributing to the so-called civilization diseases, responsible for the lowering of life quality and a large share of premature deaths in developed countries. Importantly, the phytocompounds described in this article have proven protective effects on the onset and development of civilization diseases, particularly cardiovascular disorders and diabetes. Nevertheless, it does raise the question of how their profiles and concentrations change at different development and ripening stages of CC, which is the subject of our study.

The development and ripening of fruits encompass a series of complex physiological, biochemical, and structural changes. The process affects the profile of health-promoting compounds and quality attributes, which define the technological suitability of fruits. Some compounds, for instance anthocyanins, carotenoids, and sugars, accumulate. Others, such as polyphenols in general, diminish due to dilution of the amounts accumulated at the early development stages or decompose, e.g., polysaccharides and organic acids, due to metabolic pathways characteristic of each species [24,25,26,27,28,29]. 

Various uses of CC fruits at different maturity stages have been proved earlier. For instance, fully grown but still green fruits are perfect for the preparation of pickled ‘Polish olives’ [30]; before reaching full ripeness, i.e., while still on the tree, red hard fruits can be used for candies [31]. However, it is evident that for nutritional purposes CCs are usually harvested when they reach full ripeness (dark red stage) and the most desirable flavor [25], for products such as CC juices, jams, pestils, compotes, meads, fruit beers, liqueurs, meat sauce, or their addition in other mixed-fruit products [32].

Although the total polyphenolic composition and bioactive potential have already been described in ripe CC fruits, little is known about the changes in the overall chemical composition and individual bioactive compounds during fruit development and ripening, which are addressed in this paper. A similar topic was previously dedicated to other fruits, such as black chokeberry [33], blackberries [34], highbush blueberry [27], red currants [24], sweet cherries [35], *Rosa* species (rose) [36], and strawberry [37], but not cornelian cherry (*Cornus mas* L.). Recently, Park et al. [29] performed a similar study on *Cornus officinalis* Sieb. et Zucc; however, it did not include quantification of iridoids during ripening, which is presented in our research for the first time in *Cornus* species.

The main purpose of this study was to propose a novel approach for the utilization of CC by examining how the six stages of fruit development and ripening affect basic physicochemical attributes, the profile and content of health-promoting compounds, namely polyphenols (phenolic acids, hydrolyzable tannins, anthocyanins, and flavonols), iridoids, and antioxidant properties in four Polish cultivars of CC fruits. Such findings are necessary to establish the optimum harvesting date and to select the best quality and composition of fruits dedicated for functional foods, including the food components richest in the bioactive dietary phytocompounds or semi products, such as extracts, in the development of phytopharmaceuticals preventing various civilization diseases.

## 2. Materials and Methods

### 2.1. Chemicals

The following reagents and solvents were used: acetonitrile and 98–100% formic acid acquired from Merck (Darmstadt, Germany); the standards of phenolic compounds and iridoids were purchased from Extrasynthese (Genay, France); the standards of sugars and organic acids, ferrous chloride (FeCl_3_), 2,4,6-tri(2-pyridyl)-s-triazine (TPTZ), 2,2′-azino-bis(3-ethylbenzthiazoline-6-sulfonic acid) (ABTS), potassium persulfate, and 6-hydroxy-2,5,7,8-tetramethylchroman-2-carboxylic acid (Trolox) were purchased from Sigma Chemical Co. (Steinheim, Germany); methanol and hydrochloric acid were obtained from POCh (Gliwice, Poland); and hydrochloric acid, acetic acid, sodium carbonate, and sodium acetate were purchased from Chempur (Piekary Śląskie, Poland). All reagents were of analytical or HPLC grade.

### 2.2. Raw Materials

**Figure 1:**
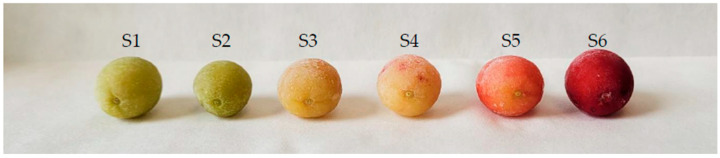
The changes in fruit color (cv. ‘Florianka’) during six studied stages.

The fruits of *C. mas* originated from Przemyśl, Poland, and were harvested in 2020, at six stages of development and ripening: on 29 June (S1), 13 July (S2), 27 July (S3), 10 August (S4), 24 August (S5), and 7 September (S6)—every two weeks (Figure 1). The following Polish cultivars (cv.) were included in this study—‘Bolestraszycki’ (early cv.), ‘Słowianin’ (mid-early cv.), ‘Paczoski’ (mid-early cv.), and ‘Florianka’ (late cv.). The relevant voucher specimens—‘Bolestraszycki’—BDPA 3951, ‘Słowianin’—BDPA 3965, ‘Paczoski’—BDPA 3966, ‘Florianka’—BDPA 1463 were deposited at the Herbarium of the Arboretum in Bolestraszyce, Poland. Precisely 80 fruits of each variety were harvested at each ripening stage and immediately frozen at −20 °C until the analysis. The flesh and skin were manually separated from stones, homogenized using a blender (Zelmer, Poland), and analyzed immediately.

### 2.3. Physicochemical Attributes

Dry weight (DW) was determined according to the gravimetric method based on the Polish Norm PN-A-75101-03:1990; briefly, the homogenous sample (ca. 2 g) was mixed with diatomaceous earth, subject to pre-drying, and final drying under reduced pressure conditions, and expressed as a percentage [%]. Titratable acidity (TA) was determined according to PN-90/A-75101/04, by titration with 0.1 N NaOH to an endpoint of pH 8.1 using an automatic titrator (pH meter type IQ 150; Warsaw, Poland), and expressed in g of malic acid per 100 g dw of the sample. Vitamin C was determined according to PN-A-04019:1998, and expressed in mg of ascorbic acid per 100 g dw of the sample. The total soluble solids (TSS) content was measured using a refractometer (Atago RX-5000, Atago Co., Ltd., Kyoto, Japan) and expressed in °Brix.

### 2.4. Samples Preparation for Analyses of Sugars and Organic Acids

An amount of 5 g of homogenized fruits with approx. 20–30 mL of distilled water was sonicated for 15 min, boiled for 30 min, transferred to 50 mL flasks with distilled water, and then centrifuged for 10 min. The supernatants (2.5 mL) were applied onto the Sep-Pak C-18 (containing 1 g of the carrier, Millipore Waters, Milford, MA, USA) and eluted with water to Eppendorf tubes. The extract was filtered through 0.45 μm Millipore filters (Waters Millipore, Milford, MA, USA).

### 2.5. Samples Preparation for Analyses of Bioactive Compounds and Antioxidant Capacity Tests

An amount of 1 g of homogenized fruits (Stages S1–S5) was weighed and extracted with 5 mL of 50% aqueous methanol (*v*/*v*), while red fruits (Stage S6) were extracted with 80% aqueous methanol (*v*/*v*), acidified with 1% HCl, and sonicated (Polsonic, Warsaw, Poland) for 15 min, at room temperature. Further, the samples were refrigerated in 4 °C for 12 h, sonicated again, and centrifuged (13,900 rpm/5 min). The supernatants were then diluted with distilled water and filtered through a PTFE 0.45 µm filter (Millex Samplicity Filter, Merck, Darmstadt, Germany) prior to HPLC-PDA, ABTS, and FRAP analysis. Samples for the UPLC-ESI-qTOF-MS/MS analysis were diluted with distilled water (1:1) and filtered through a PTFE 0.22 µm filter (Millex Samplicity Filter, Merck, Darmstadt, Germany).

### 2.6. Identification of Compounds by UPLC-ESI-qTOF-MS/MS

The compounds were tentatively identified using an Acquity ultra performance liquid chromatography system (UPLC) equipped with a quadrupole-time of flight (Q-TOF) MS instrument (UPLC/Synapt Q-TOF MS, Waters Corp., Milford, MA, USA), with an electrospray ionization (ESI) source in negative and positive modes. Separation was achieved on an Acquity BEH C18 column (100 mm × 2.1 mm i.d.; 1.7 μm) at 30 °C. The mobile phase consisted of aqueous 0.1% formic acid (A) and 100% acetonitrile (B). Samples (5 μL) were eluted according to the linear gradient described previously by Przybylska et al. [3]. The mass spectrometer operated at a source block temperature of 130 °C, desolvation temperature of 350 °C, capillary voltage of 2.5 kV, cone voltage of 30 V, and a desolvation gas (nitrogen) flow rate of 300 dm3/h, and data acquisition was in the range of *m*/*z* 100–2500 Da. Identification was based on the fragmentation patterns and by comparison with the literature data.

### 2.7. Quantification of Compounds by HPLC-PDA

#### 2.7.1. Method 1: Secondary Metabolites

The quantification of compounds was performed according to the method previously described by Przybylska et al. [3], using a Dionex (Germering, Germany) system, equipped with the diode array detector model Ultimate 3000, quaternary pump LPG-3400A, autosampler EWPS-3000SI, thermostated column compartment TCC-3000SD, and controlled by Chromeleon 7.2 software (Thermo Scientific Dionex, Sunnyvale, CA, USA). A Hypersil GOLD C18-column (250 mm × 4.6 mm i.d.; 5 μm; Thermo Fisher Scientific Inc., UK) was used for separation. The following eluents were used: C, water-FA (98.5:1.5, *v*/*v*) and D, acetonitrile-FA (98.5:1.5, *v*/*v*), and the gradient profile was as follows: 100% C, 30 min; 30% D, 33 min; 70% D, 45 min; 70% D in C, 48 min; 100% D, 55–60 min; 100% C. The flow rate of the mobile phase was 1.2 mL/min, and the injection volume was 20 μL. The column was operated at 22 °C. The UV–Vis measurements were made in the wavelength range of 200–600 nm in steps of 2 nm. Iridoids were detected at 245 nm, ellagic acid at 254 nm, hydrolyzable tannins and gallic acid at 280 nm, phenolic acids at 320 nm, flavonols at 360 nm, and anthocyanins at 520 nm and quantified using linear regression equations based on external standards. Results are provided as the mean ± standard deviation of two replications and expressed in mg/100 g of dry weight (dw) of fruits.

#### 2.7.2. Method 2: Organic Acids

Organic acids were analyzed by the HPLC method, isocratically, using 0.001 N sulfuric acid, at a 210 nm wavelength and flow rate of 0.6 mL/min. The HPLC instrument (Dionex Ultimate 3000 System, Dionex, Germering, Germany) used in this analysis was equipped with the following devices: LPG-3400A pump, EWPS-3000SI autosampler TCC-3000SD column thermostat, and the Chromeleon computer software, ver. 7.2 (Thermo Scientific Dionex, Sunnyvale, CA, USA). The separation was achieved on an Aminex HPH-87 H (300 mm × 7.8 mm i.d.) column with an IG Cation H (30 mm × 4.6 mm i.d.) precolumn from Bio-Rad (Hercules, CA, USA), at a temperature of 60 °C. Organic acids were identified by comparison with the external standards of tartaric acid, malic acid, quinic acid, and shikimic acid. Results are presented as the mean ± standard deviation of three replications and expressed in mg/100 g dw of fruit.

### 2.8. Quantification of Simple Sugars by HPLC-ELSD

Simple sugars were analyzed using an HPLC-ELSD system (Shimadzu, Tokio, Japan) equipped with an evaporative light scattering detector (ELSD) LT III. Before injection, the samples were thermostated at 10 °C, and the injection volume was 4 μL. The separation was achieved using a Shodex Asahipak NH2P-40 3E (250 mm × 3.0 mm i.d.; 4 μm) column with a Shodex Asahipak NH2P-50G 3A (10 mm × 3.0 mm i.d.; 5 μm) precolumn thermostated at 40 °C. The mobile phase was composed of water (E) and acetonitrile (F), and the gradient elution was as follows: 80–85% F; 0.80–0.90 mL/min (0–5 min), 85–87% F; 0.90–0.95 mL/min (5–7 min), 87–80% F; 0.95–0.90 mL/min (7–10 min), 80% F; 0.90–0.80 mL/min (10–16.5 min). The analysis and results were controlled by LabSolutions™ Lite software version 5.106 SP1. Compounds were identified by comparison with the external standards of glucose and fructose. Results are presented as the mean ± standard deviation of three replications and expressed in mg/100 g dw of fruit.

### 2.9. Antioxidant Capacity

The in vitro antioxidant capacity of the aqueous–methanolic extracts from cornelian cherry fruits was determined using the ABTS^•+^ (radical cation scavenging method) and FRAP (ferric reducing antioxidant power) assays, according to the methods described previously by Przybylska et al. [3]. Briefly, the samples absorbance was read at 734 nm and 593 nm for ABTS and FRAP, respectively. The results were expressed in mmol of Trolox equivalent (TE) per 100 g of dry weight of the fruit (mM TE/100 g dw). All measurements were taken on a microplate reader Synergy H1 (BioTek, Winooski, VT, USA). The analyses were run in triplicate.

### 2.10. Statistical Analysis

All statistical computations were performed with the use of Statistica Software, ver. 13.5 (StatSoft Inc., Kraków, Poland). The results were subjected to a one-factor analysis of variance (ANOVA) with the significance level *p* ≤ 0.05. Duncan’s test was used to determine significant differences between means. The results in tables are presented as means ± standard deviations.

## 3. Results and Discussion

### 3.1. Physicochemical Parameters

#### 3.1.1. Fruit Weight

As expected, the mean fruit weight gradually increased throughout the investigated period. The values differed significantly between the stages and the cultivars. At the first harvest stage (S1), ‘Paczoski’ cv. had the highest weight (0.98 g) and ‘Słowianin’ the lowest (0.84 g) (Table S1). The highest fruit weight was at S6 and ranged from 2.96 g for ‘Słowianin’ to 3.67 g for ‘Bolestraszycki’. The fruit weight showed a 3.52-fold increase on average. Other authors observed a 1.64-fold increase; however, this was in CC fruits harvested between the light yellow and dark red stages [14,24], which probably explains the discrepancies between our results and those.

#### 3.1.2. Dry Weight

Dry weight (dw) fluctuated along with the development and ripening of CC, but it generally increased in all cultivars (cvs.), except for ‘Florianka’ (Table S1). Fluctuations were also reported at different stages of black chokeberry growth by Sosnowska et al. [33]. At the initial stages (S1, S2), it had higher values and amounted to 15.91–19.99% (S1) for cvs. ‘Paczoski’ and ‘Florianka’, respectively, then it declined (from S3 to S5) to 12.89–16.32%, which was similar to the results obtained by Kucharska et al. [30] between 9.76 and 14.83% in fully grown green or light pink fruits. Furthermore, dry weight increased at S6 to reach the highest values in all cvs. except ‘Florianka’, ranging 17.85–20.77% for ‘Paczoski’ and ‘Bolestraszycki’, respectively. We suppose that ‘Florianka’ would demonstrate a similar trend, however, postponed in time, because of its status as a late-ripening cultivar. Dry weight for the cv. ‘Florianka’ amounted to 19.21% at the initial stage, then increased slightly (19.99%—S2), and dropped to reach 14.83% at S6, the lowest among all the cvs. tested. The changes in dw were statistically significant between the cvs. and the studied stages.

#### 3.1.3. Total Soluble Solids

The total soluble solids (TSS) fluctuated throughout all the studied stages (Table S1). However, the same trend was observed for all cultivars, showing higher values of the Brix refractive index in immature fruits (S1) 14.2–19.0 °Brix (‘Paczoski’ and ‘Florianka’), lower mid-ripening values, and an increase from yellow to red stages (S4–S6). The TSS of red fruits (S6) ranged 11.0–19.4 °Brix (‘Florianka’ and ‘Bolestraszycki’) and differed statistically between the cvs. The observed trend is in line with a previous study on CC by Kucharska et al. [30]—from 6.9 to 12.2 °Brix in fully grown green and light pink fruits—and by Gunduz et al. [25], in which the authors reported an increase in TSS ranging from 11.3 to 16.5 °Brix between light yellow and dark red CC, respectively. Similar trends have been observed in many other fruits, e.g., plum [38], bilberry [39], blackberry [34], or blueberry [27]. According to the literature, the value of TSS is positively correlated with sugar accumulation and it can be used as a marker of fruit maturity [40]. 

#### 3.1.4. Simple Sugars and Sweetness

The content of sugars in fruits is crucial for the sensorial properties and acceptance for consumption, and it also determines the caloric value. The contents of total sugars (TS), glucose, and fructose are depicted in Table 1. Fruit growth and ripening (S1–S6) resulted in a significant increase (mean 98.5-fold) of TS from 1.08–3.15 g/100 g dw (S1) for ‘Słowianin’ and ‘Bolestraszycki’, to 36.42–78.89 g/100 g dw (S6) for ‘Florianka’ and ‘Bolestraszycki’. At S6, the early cv. ‘Bolestraszycki’ contained the most sugars, while the late cv. ‘Florianka’ contained the least. In agreement with the reports of Kucharska [2], glucose was the main sugar in CC, accounting for 0.82–2.99 g/100 g dw at S1 (‘Słowianin’ and ‘Paczoski’) and 23.69–50.08 g/100 g dw at S6 (‘Florianka’ and ‘Bolestraszycki’). Glucose constituted 73.2–94.9% of TS at S1 and 60.6–65.0% at S6, while fructose constituted 5.4–32.3% at S1 and 35.0–39.4% at S6, respectively. The contribution of individual sugars changed considerably during the six stages studied.

In many fruits, ripening causes an accumulation of sugars and a decrease in acidity, e.g., blackberry [34], blueberry [27], and plum [38]. It is believed that the metabolism of primary sugars provides building blocks for the glycosylation of flavonoid aglycones and the accumulation of pigments. Therefore, it is suggested that the contents of 5- and 6-carbon sugars and anthocyanins increase simultaneously [39]. Additionally, the quotient of simple sugars and acids (TS/OA) is an index expressing the sweetness to sourness ratio used to classify fruits for direct consumption or for processing. In our study, this index increased steadily from 0.08–0.25 (S1) to 2.99–6.40 (S6). 

#### 3.1.5. Titratable Acidity

At the first harvest date (S1), the titratable acidity (TA) amounted to 5.36–6.59 g of malic acid/100 g dw (‘Słowianin’–‘Florianka’) and differed significantly between cvs. The development and ripening of CC caused an increase; however, only ‘Florianka’ stood out with a higher TA of 19.70 g malic acid/100 g dw, the remaining cvs. did not differ significantly (16.46–16.61 g malic acid/100 g dw). The increasing pattern was surprising compared to other fruits [41], but similar to kiwifruit [42]. This phenomenon may be associated with a higher vitamin C content or significant accumulation of malic acid (described below), contributing to the value of TA [40]. TA is a parameter that enables us to assess fruit acidity levels and predict its organoleptic properties [42]. Additionally, high acidity favors the stability of anthocyanins [41]. As shown in Table 1, the content of both parameters, TA and vitamin C, decreased at the last two stages.

#### 3.1.6. Organic Acids

Organic acids (OA), or α-hydroxycarboxylic acids, are crucial for the formation of taste characteristics in fruits. Many of them serve as approved food additives or processing aids within EU law [43]. They have also found vast applications in the cosmetic industry. The addition of acids to foods improves digestion and appetite and imparts a refreshing sensation [44]. 

Fruit growth and ripening (S1–S6) caused a significant drop (1.3-fold) in total OA for all cvs. except ‘Bolestraszycki’, ranging from 11.45–14.01 g/100 g dw (‘Bolestraszycki’ and ‘Słowianin’) to 8.96–12.17 g/100 g dw (‘Paczoski’ and ‘Florianka’), respectively (Table 1). A similar decreasing pattern was reported in plums [38]. Interestingly, the mid-early cultivars did not show significant changes between S2 and S3, and the late cv. ‘Florianka’, between S3 and S4. Significant differences were observed at S6. ‘Bolestraszycki’ was characterized by the highest OA concentration (14.83 g/100 g dw) among all stages and cultivars, followed by ‘Florianka’ (12.17 g/100 g dw), and the lowest content was determined in both ‘Słowianin’ and ‘Paczoski’ (9.02 and 8.96 g/100 g dw). 

We measured four OA—tartaric, malic, quinic, and shikimic acid. The contents of individual acids differed significantly between the cultivars and the stages studied (Table 1). Quinic acid predominated in green fruits—1.68–12.10 g/100 g dw (‘Paczoski’ and ‘Słowianin’). It is valuable for example due to its antibacterial activity [45]. The two predominant compounds at S6 were malic acid 5.50–12.24 g/100 g dw (‘Florianka’ and ‘Bolestraszycki’) and quinic acid 2.37–6.00 g/100 g dw (‘Bolestraszycki’ and ‘Florianka’); only ‘Florianka’ was characterized by higher amounts of quinic acid. It was observed that only malic acid increased (12.5–41.5-fold) towards maturity, accounting for up to 76% of total OA on average, probably due to its biosynthesis rate and this explains the increasing value of titratable acidity. Malate synthesis is mainly controlled by the activities of NAD-malate dehydrogenase (NAD-MDH, EC 1.1.1.37) and phosphoenolpyruvate carboxylase (PEPC, EC 4.1.1.31). The change from malate synthesis to its degradation can indicate the final fruit acidity [46]. Malic acid is a key contributor to acidity in numerous fruits [42] and the main organic acid of *Cornus mas* fruits, forming its tart-sour taste [2]. It is also a significant food additive (E 296), used as acidity regulator, buffering substance, aromatizer, and a preservative used in candies, drinks, jellies, fruit and vegetable preserves, and canned vegetable foodstuffs [44]. 

According to the literature, the accumulation of OA usually begins at the early development stages and provides substrates used in the respiration of a ripening fruit. OA participate in the synthesis of other compounds such as sugars, fatty acids, amino acids, and secondary metabolites during development and ripening [46,47]. However, the final content of OA depends on environmental factors and on complex metabolic pathways, i.e., the ratio of biosynthesis, degradation, and vacuolar storage, regulated by fruit enzymes and genes, and the change dynamics are typical for each species [42,46]. Moreover, the increase in air temperature and acidity are inversely dependent [24], so the increasing temperature which accompanies ripening dates can explain the OA decrease. Despite that, the role of OA in the regulation of fruit ripening has not been fully elucidated, particularly for *Cornus mas*. To our knowledge, this is the first study to assess the changes in organic acids during the development and ripening of cornelian cherry.

#### 3.1.7. Vitamin C

The content of ascorbic acid (AA) varied between the investigated stages and between cultivars. In general, we observed an increase starting from the early (S2) stages to the intermediate stages (S3–S5) and a decline at the final stage (S6) in all cvs. (Table 1). In green fruits (S1), the AA content reached 41.14–333.59 mg/100 g dw (‘Florianka’–‘Bolestraszycki’). At later dates, AA reached its peak concentration differently, depending on the cultivar: ‘Bolestraszycki’ (495.56 mg/100 g dw) and ‘Paczoski’ (408.61 mg/100 g dw) at S3, while ‘Słowianin’ (427.29 mg/100 g dw) at S4 and S5, and ‘Florianka’ (341.06 mg/100 g dw) at S4. The concentrations at S6 were significantly lower compared to S5. The lowest content was noted in the early cv. ‘Bolestraszycki’ 76.32 (mg/100 g dw) and the highest in the late cv. ‘Florianka’ (195.07 mg/100 g dw); the mid-early cvs. did not differ significantly. The highest values obtained in the intermediate stages indicate that the end of July and early August are the optimal dates of fruit harvest for the recovery of AA. A similar tendency was reported in red currants [24] and chili pepper [48], and according to [41], fruits accumulate a certain pool of AA at the green stage; however, the changes which the fruit undergoes in the course of ripening are species-dependent. For instance, in grapes, the AA value increases in veraison (the onset of the ripening of the grapes) but later decreases in pink and red maturation [24]. It increases in tomatoes [41] but decreases in peaches [28]. The ripening behavior of vitamin C may result from variations in biosynthesis rate and metabolic pathways, e.g., the activity of enzymes that enhance its oxidation [41]. Contrastingly, a decrease in vitamin C may also result from fruit growth, not the degradation of this compound [49].

### 3.2. Bioactive Secondary Metabolites

#### 3.2.1. General

**Figure 2:**
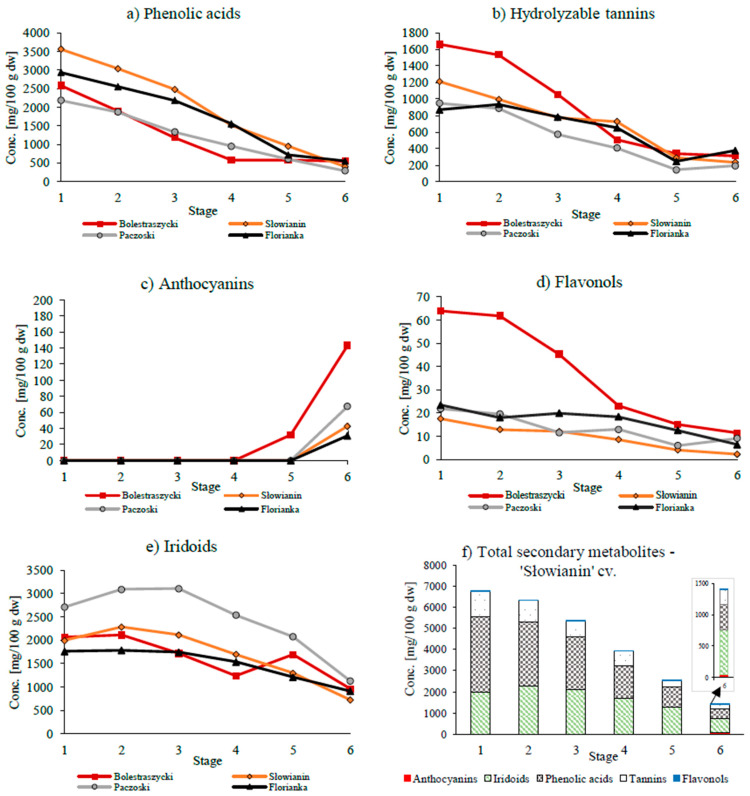
Changes dynamics of phenolic acids (**a**), hydrolyzable tannins (**b**), anthocyanins (**c**), flavonols (**d**), iridoids (**e**), and total secondary metabolites—‘Słowianin’ cv. (**f**). The concentrations (Conc.) are expressed as mg/100 g dw and the numbers (1–6) on the horizontal axis represent the studied stages. For the homogenous groups (Duncan’s test), see Supplementary File Tables S3–S5.

The highest mean concentrations of total secondary metabolites were observed at S1 for ‘Słowianin’—6779.62 mg/100 g dw (Figure 2) and the lowest for ‘Florianka’—5589.07 mg/100 g dw (Figure S1). A significant decrease occurred during ripening (S1–S6). At S6, the ‘Bolestraszycki’ cv. was the most abundant in bioactive phytocompounds—1974.59 mg/100 g dw—while ‘Słowianin’ was the least—1403.67 mg/100 g dw (Table S3). In summary, unripe fruits (S1) were several times (3.0–4.8) richer in these compounds, which is similar to previous findings for CC [25,30], blueberry [27], and peach [28]. However, qualitative identification by means of UPLC-ESI-qTOF-MS/MS yielded a similar profile of the secondary metabolites at S1–S6 (Table S2).

To evaluate the contribution of each class, we grouped the compounds as polyphenols (phenolic acids, hydrolyzable tannins, anthocyanins, flavonols) and iridoids. Phenolic acids (37.3–52.6%), followed by iridoids (29.4–46.2%), tannins (9.9–16.4%), and flavonols (0.3–1.0%) dominated in unripe fruits (S1). During ripening (S1–S6), the mean concentrations of the evaluated classes decreased as follows: phenolic acids 5.1-fold, flavonols 4.3-fold, hydrolysable tannins 4.2-fold, and iridoids 2.3-fold, while anthocyanins increased 56.9-fold (Figure 2). The composition of ripe fruits (S6) changed considerably, but iridoids, phenolic acids, and tannins remained the main compounds, and anthocyanins appeared as additional constituents only at this stage. Iridoids predominated (48.5–67.2%), followed by phenolic acids (17.0–29.5%), tannins (11.6–20.0%), anthocyanins (1.6–7.3%), and flavonols (0.2–0.6%) (Figure 2 and Table S3).

#### 3.2.2. Polyphenols

Polyphenols and terpenoids protect fruits against pathogens, frugivores, and environmental stress and contribute to color and flavor [50,51]. Flavonoids mediate pollen formation and hence plant reproduction. Polyphenols are the determinants of the health-promoting value of fruits [24,26]. However, increasing water content in cells and decreasing polyphenol biosynthesis (lower activity of phenylalanine ammonia-lyase, EC 4.3.1.5) during ripening affect lower concentrations at later stages [28,52]. The total phenolic content (phenolic acids, hydrolysable tannins, anthocyanins, and flavonols) was higher at S1 (3155.34–4789.78 mg/100 g dw, ‘Paczoski’ and ‘Słowianin’) and 4–7 times lower at S6 (550.50–1017.74 mg/100 g dw, ‘Paczoski’ and ‘Bolestraszycki’) (Table S3). In general, ‘Paczoski’ was the poorest source of polyphenols.

##### Phenolic Acids

Phenolic acids (PhAs) are commonly found in edible plants and are appreciated for numerous biological activities, especially as antioxidants [52]. They impart sourness, bitterness, and astringency to many foods of plant origin [53].

The experiment showed that PhAs concentrations varied among the cultivars (Figure 2 and Table S3). Unripe fruits were 4.7–8.8-fold richer in PhAs than ripe fruits, amounting to 2185.29–3561.64 mg/100 g dw (‘Paczoski’ and ‘Słowianin’) at S1, and 285.37–552.19 mg/100 g dw (‘Paczoski’ and ‘Florianka’) at S6, respectively. We identified six hydroxycinnamic acids (Table S4). The caftaric acid *cis*-isomer (caffeic acid bond to tartaric acid) dominated in all cvs., both in unripe fruits (37.7–68.3% PhA) and ripe fruits (36.0–69.1% PhA). The second dominant compound was the caftaric acid *trans*-isomer (12.8–20.3% (S1) and 15.1–36.8% (S6) PhA) or coutaric acid *cis*-isomer (14.4–42.0% (S1) and 8.5–32.9% (S6) PhA), depending on the cultivar. The remaining PhAs were the coutaric acid *trans*-isomer, free *p*-coumaric acid, and *p*-coumaric acid derivative. The mean PhAs content decreased 5.1 times, but they remained the predominant phenolic compounds at S6. Our results are in agreement with previous findings reporting a decrease in PhAs content during ripening [24,39].

##### Hydrolyzable Tannins

Tannins are one of the defensive compounds of plants, but due to their low toxicity (compared to alkaloids and terpenoids), they accumulate in substantial amounts, even up to 10% dw, to protect the fragile parts against frugivores, parasites, or microorganisms [54]. In our earlier work, we demonstrated that both fruits and stones [2,3,4] are abundant in monomeric, dimeric, and trimeric derivatives of gallic acid (GA) and ellagic acid (EA). 

As far as we are concerned, in the present study we identified and quantified hydrolyzable tannins (HTs) for the first time at different ripening stages of CC. HTs, esterified forms of GA and EA, were the third predominant bioactive compounds at S1 (Figure 2 and Table S3). The unripe fruits (S1) provided more HTs, 869.74–1661.23 mg/100 g dw (‘Florianka’-‘Bolestraszycki’), and a progressive drop (4.2-fold) was observed until the final content (S6) of 194.29–376.11 mg/100 g dw (‘Paczoski’ and ‘Florianka’). Gunduz et al. [25] also reported higher content of tannins in yellow CC fruit (0.45% fw) and a lower content (0.16% fw) in dark red fruit, but determined by a spectrophotometric method and expressed as tannic acid. The same tendency for HTs, GA, and EA was reported for blackberries [34] and Kakadu plums [40]. 

A total of 29 HTs were tentatively identified in the experiment. The qualitative profile of HTs was similar in unripe and ripe fruits, but higher contents of more complex HTs, i.e., dimeric ellagitannin (ET) camptothin A (27.7–30.9%) and cornusiin A (16.4–26.4%) and trimeric ETs cornusiin F (12.2–13.6%) and cornusiin C (6.8–14.0%) were observed in unripe fruits and decreased consecutively (Tables S2 and S5). The predominant compounds (S1) were camptothin A—253.39–477.55 mg/100 g dw—and cornusiin A—143.02–350 mg/100 g dw (‘Florianka’ and ‘Bolestraszycki’), in contrast to the CC stones which contained more cornusiin C and F [3]. As ripening progressed, the share of dimeric and trimeric ETs decreased, while the share of gallotannins (mainly mono-*O*-gall-*β*-D-glucose) increased (S1–S6) from 2.1–14.5% to 14.4–50.0% (‘Paczoski’ and ‘Bolestraszyki’). We also observed lower amounts of GA and EA. Gallic acid decreased gradually from 37.60–91.39 mg/100 g dw (‘Paczoski’ and ‘Florianka’) at S1 to 0.00–10.32 mg/100 g dw (‘Słowianin’ and ‘Florianka’) at S6. Regarding EA, a slight increase was observed at the intermediate stages, followed by a further drop from 1.16–9.33 mg/100 g dw (‘Słowianin’ and ‘Florianka’) at S1 to 0.94–2.76 mg/100 g dw (‘Słowianin’ and ‘Florianka’) at S6, and the highest concentration of 31.62 mg/100 g dw was observed for ‘Florianka’ at S3.

The lower content of tannins in ripe fruits could be explained by their polymerization and binding to proteins, polysaccharides, or fiber [40]. Tannins precipitate salivary proteins, which is reflected in an unpleasant astringent feeling, and high concentrations are not desired in foods. On the other hand, they interact with digestive enzymes and reduce the absorption of sugar and lipids from the digestive tract, which is a potential antidiabetic and antihyperlipidemic trait [53,55]. Some of the HTs present in CC showed antiviral activity against herpes simplex virus type 1 [56] and antibacterial action [57]. That said, HTs deserve more attention in future studies, as they have not been widely examined in CC.

##### Anthocyanins

At green and yellow stages, there were no anthocyanins in CC. Anthocyanin accumulation started only at the last stage, which is consistent with a recent study on *Cornus officinalis* fruits [29] and indicates the time point of color development (Figure 1) [27,47,58]. Figure 2 and Table S3 show that the intensity of the time-dependent increase in anthocyanins varies between cvs. As expected, the biggest accumulation was reported in the early cv. ‘Bolestraszycki’ (143.17 mg/100 g dw) and the lowest in the late cv. ‘Florianka’ (30.83 mg/100 g dw). The average anthocyanin content in fruits was 56.87 mg/100 g dw. We quantified four anthocyanins (Table S4): cyanidin 3-*O*-galactoside, cyanidin 3-*O*-robinobioside, pelargonidin 3-*O*-galactoside, and pelargonidin 3-*O*-robinobioside in each studied cultivar. Delphinidin 3-*O*-galactoside, a minor anthocyanin of CC, was not detected. The concentration of anthocyanins at S6 differed between cultivars, but pelargonidin 3-*O*-galactoside predominated, with the content of 22.03–74.29 mg/100 g dw in ‘Florianka’ and ‘Bolestraszycki’, representing 71.4% and 51.9% of total anthocyanins, respectively. Earlier research [59] also showed that pelargonidin 3-*O*-galactoside dominated in the same CC cultivars.

Anthocyanins are powerful antioxidants and antidiabetic molecules [60,61]. Anthocyanins of CC show anti-atherosclerotic effects [8,23]; therefore, it is optimal to select fully ripe, dark red CM fruits for the isolation of these compounds and for consumption. Under the conditions of our study, ‘Bolestraszycki’ appears to be the most prominent source of anthocyanins among the cultivars tested.

##### Flavonols

Among flavonols, we identified only one compound, namely quercetin 3-*O*-glucuronide, in all cultivars (Figure 2, Tables S3 and S4). ‘Bolestraszycki’ stood out with its particularly high content (63.89 mg/100 g dw) at the first stage (S1), while other cultivars contained similar concentrations from 17.55 to 23.46 mg/100 g dw (‘Słowianin’ and ‘Florianka’). Even lower values were determined at S6, falling within the range of 4.09–11.43 mg/100 g dw (‘Słowianin’ and ‘Bolestraszycki’), which corresponds to earlier reports for quercetin 3-*O*-glucuronide in bilberry [39]. Flavonols were one of the least prevalent secondary metabolites found in CC, comprising only 0.2 to 1.0% of this fraction.

#### 3.2.3. Iridoids

Iridoids are heterocyclic monoterpenoids, which are infrequent in edible fruits, but are present in certain species such as CC, olives, noni, cranberries, or blueberries. They show many bioactivities, mainly anti-inflammatory, antiglaucomic, antidiabetic, and neuroprotective [32]. As far as we are concerned, the literature lacks information on iridoids in developing and ripening fruits, and specifically in CC. Iridoids were the second most abundant class of bioactive compounds at S1, and the most abundant at S6 (Figure 2, Table S3). The concentrations varied among the cultivars. In general, unripe fruits (S1) contained more iridoids than ripe fruits (S6), in the range of 1758.94–2709.88 mg/100 g dw (‘Florianka’ and ‘Paczoski’) and 721.15–1126.19 mg/100 g dw (‘Słowianin’ and ‘Paczoski’), respectively. All cultivars contained three iridoids, loganic acid, and cornuside, and traces of loganin. The total quantified iridoids showed a slight increase from S1 to S3, and a significant decrease at the subsequent stages. CC contained the highest concentrations of iridoids at S2 1780.09–2282.50 mg/100 g dw (‘Florianka’ and ‘Słowianin’), or S3 3108.56 mg/100 g dw (‘Paczoski’). At the last stage, iridoids were present in amounts from 721.15 to 1126.19 mg/100 g dw (‘Słowianin’ and ‘Paczoski’). Loganic acid was the main iridoid (Table S4), which is in agreement with previous studies by Kucharska [2,59], and comprised 68.6–84.7% in green fruits (S1) and 91.7–93.8% in red fruits (S6), while cornuside comprised 15.3–31.4% and 6.17–8.26%, respectively. During ripening (S1–S6), the content of iridoids decreased to the smallest extent (2.3 times) compared to the remaining secondary metabolites. Additionally, loganic acid turned out to be more stable than cornuside, showing an average decrease of 1.74 times, while cornuside decreased 6.13 times (S1–S6). To the best of our knowledge, this work is the first to quantify the changes in iridoids during the ripening of CC. Many studies have shown that iridoid-rich fruits or iridoids can be used to design novel foods with unique organoleptic and functional properties (e.g., ciders, beers, meads, smoothies, pickles, yogurts). A good example is an iridoid-enriched chocolate spread that reduces blood glycemia in diabetic patients and provides polyphenols and vitamin E [32].

### 3.3. Antioxidant Capacity

**Figure 3:**
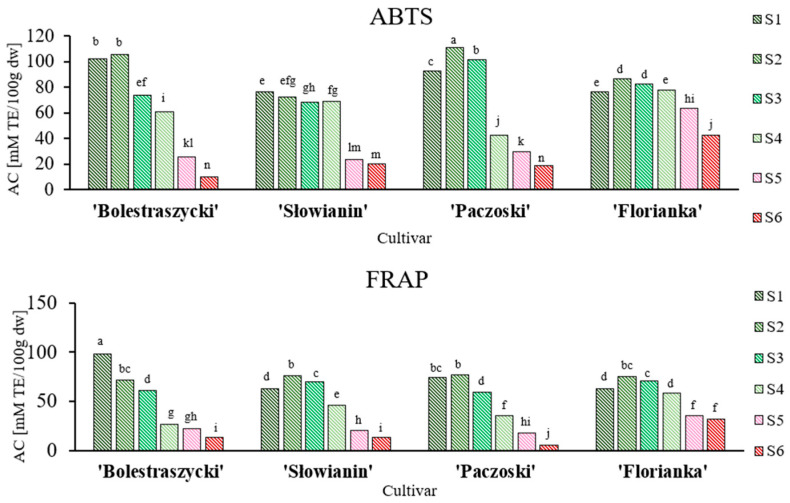
Changes in antioxidant capacity (AC) by ABTS and FRAP assays (mM TE/100 g dw) during six developmental and ripening stages (S1–S6) of CC and between cultivars. Colors of the bars are symbolic and represent the transition from green to red. The letters a–n stand for homogenous groups (Duncan’s test).

The antioxidant capacity of fruits (S1–S6) was determined using the ABTS and FRAP methods (Figure 3 and Table S3). Unripe fruits were characterized by a significantly higher antioxidant capacity (6.3 times on average) in both assays, ranging from 76.31–102.12 mM TE/100 g dw (‘Słowianin’ and ‘Bolestraszycki’) at S1 to 9.78–42.40 mM TE/100 g dw (‘Bolestraszycki’ and ‘Florianka’) at S6 for ABTS, and from 62.76–97.76 mM TE/100 g dw (‘Florianka’ and ‘Bolestraszycki’) at S1 to 5.32–31.73 mM TE/100 g dw (‘Paczoski’ and ‘Florianka’) at S6 for FRAP. The antioxidant capacity and polyphenol content are generally correlated and decrease simultaneously [25,28,62]. According to the literature, the polyphenol content of CC significantly affects the antioxidant capacity [63], which was also observed in this study (Figure 2 and Figure 3).

## 4. Conclusions

Ripe cornelian cherry (CC) is known for its rich composition of bioactive substances, particularly those with antioxidant, anti-inflammatory, and antidiabetic properties. This study showed that the chemical composition of CC fruits is significantly affected by the ripening process, as well as the cultivar.

Our results showed that unripe fruits were the richest in secondary metabolites, such as phenolic acids, hydrolyzable tannins (mainly dimeric and trimeric), iridoids, and flavonols; as well as organic acids. Phenolic acids (37.3–52.6%), iridoids (29.4–46.2%), and tannins (9.9–16.4%) predominated in unripe fruits. All the groups underwent a considerable decrease along with the antioxidant capacity, but iridoids were affected to the lowest extent among secondary metabolites. It was shown that hydrolyzable tannins (HTs) contributed to the substantial proportion of polyphenols in unripe (22.7–38.5%) and ripe fruits (31.3–38.9%), and to the knowledge of the authors, it was the first report on the composition and ripening changes in HTs in CC. On the other hand, the highest content of anthocyanins, sugars, and malic acid was reported in ripe fruits. In ripe fruits, the main secondary metabolites were iridoids (48.5–67.2%), phenolic acids (17.0–29.5%), tannins (11.6–20.0%), and anthocyanins (1.6–7.3%). Interestingly, the intermediate stages were characterized by the highest content of vitamin C, ellagic acid, gallotannins, and loganic acid. Despite the increase in malic acid, and the titratable acidity, the ratio of sugars to acids was highest in ripe fruits, which is favorable for nutritional uses. 

The metabolic behavior of the individual compounds and the groups of compounds was revealed for the first time, providing a new approach for the utilization of cornelian cherry fruits. Understanding those changes at different degrees of ripening enables the choice of the most accurate composition and properties of CC. Unripe fruits are an excellent source of bioactive molecules, richer than ripe fruits, which makes them a highly valuable resource for the design of dietary supplements for prophylaxis or natural-origin pharmaceuticals. Therefore, the application of green cornelian cherry should be investigated in the future, and this study provided a comprehensive assessment which can be advantageous for further studies in this field. Nonetheless, ripe CC still provides bioactive compounds in different concentrations or with slightly different profiles, but at the same time, it provides dietary antioxidants and is a good raw material for new functional foods.

## Figures and Tables

**Table 1:** The changes in basic physicochemical attributes during the development and ripening of cornelian cherry fruits.

	Stage	Glucose	Fructose	TS [g/100 g dw]	Tartaric Acid	Malic Acid	Quinic Acid	Shikimic Acid	OA [g/100 g dw]	TA [g MA/100 g dw]	AA [mg/100 g dw]	TS/OA
Bol	S1	1.65 ± 0.08 ^j^	0.22 ± 0.02 ^m^	1.86 ^p^	0.25 ± 0.06 ^fghi^	0.55 ± 0.07 ^jkl^	10.24 ± 0.55 ^de^	0.40 ± 0.00 ^i^	11.45 ^ghi^	6.16 ± 0.00 ^jk^	333.59 ± 1.97 ^e^	0.16 ^l^
	S2	2.51 ± 0.15 ^ij^	0.55 ± 0.06 ^m^	3.06 ^po^	0.17 ± 0.02 ^hi^	1.05 ± 0.10 ^jk^	7.90 ± 0.67 ^g^	0.44 ± 0.02 ^hi^	9.56 ^jk^	7.37 ± 0.18 ^i^	457.30 ± 10.00 ^b^	0.32 ^jkl^
	S3	4.56 ± 0.09 ^hi^	1.12 ± 0.03 ^lm^	5.68 ^no^	0.13 ± 0.00 ^i^	2.28 ± 0.01 ^hi^	8.41 ± 0.01 ^fg^	0.32 ± 0.00 ^j^	11.15 ^hi^	10.53 ± 0.23 _g_	495.56 ± 26.61 ^a^	0.51 ^ijk^
	S4	10.25 ± 0.24 ^fg^	3.36 ± 0.41 ^jk^	13.62 ^jk^	0.09 ± 0.00 ^i^	5.45 ± 0.17 ^f^	4.26 ± 0.20 ^i^	0.14 ± 0.00 ^m^	9.94 ^ijk^	13.18 ± 0.71 ^e^	402.11 ± 26.91 ^cd^	1.37 ^g^
	S5	11.34 ± 1.43 ^f^	5.88 ± 0.58 ^gh^	17.22 ^hi^	0.13 ± 0.01 ^i^	9.23 ± 0.95 ^b^	2.29 ± 0.29 ^jk^	0.19 ± 0.03 ^l^	11.85 ^efgh^	17.24 ± 0.55 ^bc^	257.16 ± 2.98 ^g^	1.45 ^fg^
	S6	50.80 ± 1.64 ^a^	28.09 ± 0.95 ^a^	78.89 ^a^	0.20 ± 0.00 ^ghi^	12.24 *±* 0.81 ^a^	2.37 ± 0.44 ^jk^	0.01 ± 0.00 ^o^	14.83 ^abc^	16.46 ± 0.03 ^cd^	76.32 ± 1.87 ^k^	5.32 ^c^
Słow	S1	0.82 ± 0.04 ^j^	0.26 ± 0.00 ^m^	1.08 ^p^	1.01 ± 0.00 ^c^	0.36 ± 0.00 ^kl^	12.10 ± 0.02 ^b^	0.54 ± 0.00 ^e^	14.01 ^bcd^	6.59 ± 0.14 ^ij^	131.56 ± 1.77 ^j^	0.08 ^l^
	S2	1.46 ± 0.03 ^j^	1.05 ± 0.06 ^lm^	2.51 ^p^	0.91 ± 0.00 ^c^	1.16 ± 0.00 ^j^	10.68 ± 0.01 ^cd^	0.64 ± 0.00 ^d^	13.38 ^cde^	6.49 ± 0.05 ^ijk^	190.06 ± 8.14 ^i^	0.19 ^l^
	S3	5.28 ± 0.73 ^h^	3.50 ± 0.33 ^jk^	8.78 ^m^	0.64 ± 0.07 ^d^	2.20 ± 0.06 ^hi^	9.82 ± 0.68 ^de^	0.68 ± 0.03 ^d^	13.34 ^cde^	8.90 ± 0.04 ^h^	319.61 ± 9.67 ^ef^	0.66 ^i^
	S4	10.41 ± 1.13 ^fg^	4.70 ± 0.89 ^hij^	15.11 ^ji^	0.36 ± 0.02 ^efgh^	4.90 ± 0.06 ^f^	5.87 ± 0.11 ^h^	0.46 ± 0.01 ^fg^	11.59 ^fgh^	13.17 ± 0.54 ^e^	427.29 ± 28.46 ^c^	1.30 ^g^
	S5	17.74 ± 0.85 ^d^	10.17 ± 2.28 ^e^	27.92 ^e^	0.28 ± 0.01 ^fghi^	7.19 ± 0.39 ^de^	2.88 ± 0.09 ^j^	0.26 ± 0.01 ^k^	10.62 ^hij^	15.80 ± 1.75 ^d^	425.77 ± 18.15 ^c^	2.63 ^e^
	S6	34.57 ± 4.20 ^b^	19.85 ± 0.87 ^c^	54.42 ^c^	0.12 ± 0.00 ^i^	7.79 ± 0.16 ^cd^	1.07 ± 0.01 ^l^	0.03 ± 0.00 ^o^	9.02 ^k^	16.53 ± 0.30 ^cd^	167.26 ± 11.25 ^i^	6.03 ^b^
Pacz	S1	2.99 ± 0.11 ^hij^	0.17 ± 0.01 ^m^	3.15 ^po^	0.53 ± 0.00 ^def^	0.65 ± 0.14 ^jkl^	1.68 ± 0.23 ^cde^	1.29 ± 0.05 ^b^	12.94 ^defg^	6.50 ± 0.08 ^ijk^	168.36 ± 9.22 ^i^	0.24 ^kl^
	S2	5.41 ± 0.03 ^h^	4.25 ± 0.00 ^ij^	9.66 ^lm^	0.41 ± 0.07 ^efg^	1.86 ± 0.34 ^i^	1.47 ± 0.04 ^ef^	1.43 ± 0.00 ^a^	13.12 ^def^	6.69 ± 0.06 ^ij^	336.32 ± 24.71 ^e^	0.74 ^i^
	S3	7.97 ± 0.37 ^g^	5.34 ± 0.03 ^ghi^	13.31 ^jk^	0.37 ± 0.07 ^efgh^	3.87 ± 0.44 ^g^	1.07 ± 0.07 ^g^	1.03 ± 0.07 ^c^	13.04 ^def^	10.59 ± 0.06 ^g^	408.61 ± 9.04 ^cd^	1.03 ^h^
	S4	10.63 ± 1.11 ^f^	7.42 ± 1.04 ^f^	18.05 ^h^	0.26 ± 0.05 ^fghi^	6.55 ± 0.21 ^e^	0.44 ± 0.05 ^ij^	0.52 ± 0.04 ^e^	10.73 ^hij^	14.15 ± 1.05 ^e^	392.69 ± 0.77 ^d^	1.68 ^f^
	S5	17.83 ± 0.25 ^d^	11.92 ± 0.66 ^d^	29.75 ^e^	0.17 ± 0.01 ^hi^	9.15 ± 0.61 ^b^	0.18 ± 0.00 ^kl^	0.40 ± 0.02 ^i^	11.08 ^hij^	18.06 ± 0.65 ^b^	395.11 ± 4.63 ^d^	2.69 ^e^
	S6	34.74 ± 0.83 ^b^	22.57 ± 0.64 ^b^	57.31 ^b^	0.09 ± 0.00 ^i^	8.18 ± 0.02 ^c^	0.11 ± 0.00 ^l^	0.08 ± 0.00 ^n^	8.96 ^k^	16.61 ± 0.36 ^cd^	177.28 ± 14.13 ^i^	6.40 ^a^
Flor	S1	2.29 ± 0.18 ^ij^	1.01 ± 0.00 ^lm^	3.13 ^po^	1.86 ± 0.17 ^a^	0.13 ± 0.00 ^l^	10.81 ± 0.02 ^cd^	0.41 ± 0.00 ^i^	13.20 ^de^	5.36 ± 0.01 ^k^	41.14 ± 2.16 ^l^	0.25 ^kl^
	S2	5.39 ± 0.10 ^h^	2.67 ± 0.09 ^k^	8.06 ^mn^	1.53 ± 0.21 ^b^	0.53 ± 0.08 ^jkl^	11.74 ± 0.06 ^bc^	0.49 ± 0.00 ^efg^	14.28 ^abcd^	6.05 ± 0.04 ^jk^	133.68 ± 0.48 ^j^	0.56 ^ij^
	S3	9.67 ± 0.22 ^fg^	2.31 ± 0.08 ^kl^	11.98 ^kl^	1.08 ± 0.00 ^c^	1.02 ± 0.01 ^jk^	12.63 ± 0.02 ^ab^	0.49 ± 0.00 ^ef^	15.23 ^ab^	6.38 ± 0.14 ^ijk^	222.17 ± 5.32 ^h^	0.79 ^hi^
	S4	14.98 ± 0.55 ^e^	6.67 ± 0.11 ^fg^	21.66 ^g^	0.96 ± 0.31 ^c^	0.76 ± 0.04 ^jkl^	13.27 ± 1.66 ^a^	0.34 ± 0.04 ^j^	15.33 ^ab^	7.53 ± 0.06 ^i^	341.06 ± 8.86 ^e^	1.43 ^fg^
	S5	18.56 ± 0.15 ^d^	6.47 ± 0.36 ^fg^	25.02 ^f^	0.87 ± 0.01 ^c^	2.74 ± 0.14 ^h^	11.76 ± 0.42 ^bc^	0.22 ± 0.01 ^l^	15.59 ^a^	12.06 ± 0.55 ^f^	298.49 ± 9.38 ^f^	1.61 ^fg^
	S6	23.69 ± 1.40 ^c^	12.73 ± 0.80 ^d^	36.42 ^d^	0.55 ± 0.01 ^de^	5.50 ± 0.02 ^f^	6.00 ± 0.08 ^h^	0.12 ± 0.00 ^mn^	12.17 ^efgh^	19.70 ± 0.07 ^a^	195.07 ± 19.46 ^hi^	2.99 ^d^

Legend: The values are expressed as means ± SD, n = 3; small letters stand for homogenous groups (Duncan’s test); Bol, ‘Bolestraszycki’; Słow, ‘Słowianin’; Pacz, ‘Paczoski’; Flor, ‘Florianka’, S1–S6 development and ripening stages; TS, total sugars; TA, titratable acidity OA, total organic acids; AA, ascorbic acid; SD, standard deviation.

## Data Availability

Data is contained within the manuscript.

## Supplementary Materials

The following supporting information can be downloaded at: https://www.mdpi.com/article/10.3390/antiox13020229/s1, Table S1: The changes in color, fruit weight, dry weight, and TSS during the development and ripening of cornelian cherry fruits; Table S2: UPLC-ESI-qTOF-MS/MS identification of the secondary metabolites in cornelian cherry fruits; Table S3: HPLC-PDA quantification of the secondary metabolites and antioxidant capacity during the development and ripening of cornelian cherry fruits; Table S4: HPLC-PDA quantification [mg/100 g dw] of the individual polyphenols during the development and ripening of cornelian cherry fruits; Table S5: HPLC-PDA quantification [mg/100 g dw] of the individual hydrolysable tannins and iridoids during the development and ripening of cornelian cherry fruits; Figure S1: Changes in the content (mg/100 g dw) of all groups of analyzed secondary metabolites during the development and ripening of cornelian cherry.
