# Supplementary material for: The Health-Promoting Quality Attributes, Polyphenols, Iridoids and Antioxidant Activity during the Development and Ripening of Cornelian Cherry (Cornus mas L.)
Source: Antioxidants (Basel). 2024 Feb 13;13(2):229. doi: 10.3390/antiox13020229 (PMC10885943; doi:10.3390/antiox13020229)
Supplement: Supplementary file 1 [file antioxidants-13-00229-s001.zip › antioxidants-2780615-supplementary.pdf]

Table S1 The changes of color, fruit weight, dry weight, and TSS during the development and ripening of cornelian cherry fruits.

Stage	Color	Fruit weight [g] *	Dry weight [%]	TSS [°Brix]
**Cultivar**		$\bar{x}$	*mean ± SD*	$\bar{x}$
**'Bolestraszycki'**				
S1	green	0.90 [v]	18.86 ± 0.0 [e]	18.5 [a]
S2	green	1.03 [r]	19.15 ± 0.5 [cd]	16.3 [c]
S3	green	1.51 [l]	16.32 ± 0.4 [h]	13.1 [h]
S4	yellow with light blush	2.42 [g]	15.21 ± 0.8 [i]	11.7 [k]
S5	light red	2.78 [e]	12.89 ± 0.4 [m]	14.7 [p]
S6	red	3.67 [a]	20.77 ± 0.0 [a]	19.4 [a]
**'Słowianin'**				
S1	green	0.84 [w]	17.15 ± 0.4 [g]	14.8 [f]
S2	green	0.93 [u]	17.40 ± 0.1 [fg]	11.8 [j]
S3	green	1.08 [p]	15.72 ± 0.1 [i]	11.9 [i]
S4	green	1.49 [m]	14.73 ± 0.1 [j]	11.6 [l]
S5	green/yellow	2.26 [h]	13.6 ± 0.2 [kl]	10.1 [o]
S6	light red/red	2.96 [d]	19.63 ± 0.1 [bc]	16.1 [d]
**'Paczoski'**				
S1	green	0.98 [s]	15.91 ± 0.2 [hi]	14.2 [g]
S2	green	1.03 [r]	15.56 ± 0.1 [i]	9.1 [s]
S3	green	1.48 [n]	13.74 ± 0.1 [k]	10.8 [n]
S4	yellow	2.07 [j]	12.94 ± 0.0 [m]	9.3 [r]
S5	yellow with light blush	2.74 [f]	13.04 ± 0.1 [lm]	9.6 [q]
S6	light red/red	3.26 [b]	17.85 ± 0.1 [f]	14.9 [e]
**'Florianka'**				
S1	green	0.94 [t]	19.21 ± 0.0 [cd]	19.0 [b]
S2	green	1.04 [q]	19.99 ± 0.1 [b]	7.2 [u]
S3	green	1.22 [o]	19.55 ± 0.3 [bc]	8.8 [t]
S4	green/yellow	1.61 [k]	18.45 ± 0.2 [e]	9.6 [q]

S5	yellow	2.14 [i]	14.53 ± 0.3 [j]	11.8 [j]
S6	light red/red	3.01 [c]	14.83 ± 0.4 [j]	11.0 [m]

Legend: S1-S6 development and ripening stages; *mean fruit weight, n = 80; TSS, total soluble solids; SD, standard deviation;

Table S2 UPLC-ESI-qTOF-MS/MS identification of the secondary metabolites in cornelian cherry fruits.

No.	tR (min)	Λmax	Tentative identification (isomer no.)	MS1 [M – H]– (m/z)	MS2 other ions (m/z)
			HYDROLYSABLE TANNINS		
1	1.51	277	Mono-*O*-galloyl-*β*-D-glucose (1)	331	169, 125
2	1.82	271	Gallic acid	169	125
3	1.78	266	Gemin D (1)	633	301, 275, 249, 169, 125
4	2.27	256	Gemin D (2)	633	301, 275, 249, 169, 125
5	2.66	275	Di-*O*-galloyl-*β*-D-glucose (1)	483	331, 169, 125
6	2.72	219, 262	Bis-HHDP-hexoside (2)	783	481, 331, 301, 275, 169, 125
7	3.03	219, 264	Camptothin A (1)	1417, 708–2	1247, 1115, 785, 765, 633, 613, 451, 301, 275, 169, 125
8	3.44	219, 264	Camptothin A (2)	1417, 708–2	1247, 1115, 785, 765, 633, 613, 451, 301, 275, 169, 125
9	3.68	219, 262	Bis-HHDP-hexoside (2)	783	633, 481, 301, 275, 249
10	3.88	219, 262	Camptothin A (3)	708–2, 1417	1247, 935, 785, 765, 633, 613, 451, 301, 275, 249, 169
11	3.91	219, 263	Cornusiin F (1)	2201, 1100–2	933, 785, 783, 633, 451, 425, 301, 275, 249, 169, 125
12	4.13	219, 263	Cornusiin F (2)	2201, 1100–2	1567, 1247, 933, 785, 765, 633, 613, 451, 483, 301, 249, 169, 125
13	4.41	219, 269	Cornusiin A (1)	1569, 784–2	1417, 1247, 935, 785, 765, 633, 451, 301, 275, 249, 169, 125
14	4.55	219, 262	Cornusiin F (3)	2201, 1100–2	2031, 1569, 1417, 1247, 933, 785, 765, 633, 613, 483, 451, 313, 301, 275, 271, 249, 169, 125
15	4.75	219, 269	Cornusiin A (2)	1569, 784–2	1085, 933, 785, 765, 633, 451, 331, 301, 275, 271, 249, 169, 125
16	4.79	219, 269	Cornusiin F (4)	2201, 1100–2	1569, 1247, 1015, 933, 785, 765, 665, 633, 613, 451, 483, 451, 301, 275, 249, 169, 125
17	4.83	219, 270	Tri-*O*-galloyl-*β*-D-glucose (1)	635	313, 169, 125
18	4.94	219, 270	Tellimagrandin I	785	765, 633, 483, 451, 313, 301, 275, 249, 169, 125
19	5.05	219, 269	Cornusiin C (1)	2353, 1176–2	2201, 1569, 933, 785,765, 633, 465, 451, 301, 275, 249, 169, 125
20	5.24	219, 267	Cornusiin A (3)	1569, 784–2	935, 785, 765, 633, 465, 451, 301, 275, 249, 169, 125
21	5.49	219, 269	Cornusiin A (4)	1569, 784–2	1249, 935, 785, 765, 633, 613, 483, 451, 331, 313, 301, 275, 249, 169, 125
22	5.57	219, 270	Cornusiin C (2)	2353, 1176–2	1569, 1247/1249, 933, 785, 765, 633, 451, 301, 275, 249, 169, 125

23	5.65	219, 269	Cornusiin C (3)	2353, 1176–2	1569, 1417, 935, 785, 768, 633, 451, 331, 301, 275, 249, 169, 125
24	5.72	219, 267	Cornusiin A (5)	1569, 784–2	935, 785, 765, 633, 483, 451, 425, 301, 275, 169, 125
25	5.87	219, 279	Corn D or Cmpt B (1)	1721, 860–2	1569, 1417, 935, 785, 633, 301, 275, 249, 169, 125
26	5.94	219, 279	Corn D or Cmpt B (2)	1721, 860–2	1569, 1417, 937, 785, 765, 633, 451, 301, 275, 249, 169, 125
27	6.22	219, 269	Cornusiin A (6)	1569, 784–2	141, 935, 785, 765, 633, 483, 451, 425, 331, 301, 275, 249, 169, 125
28	6.38	219, 269	Cornusiin C (4)	2353, 1176–2	1569, 1417, 937, 785, 633, 613, 451, 301, 275, 249, 169, 125
29	6.47	254, 360	Ellagic acid	301	275, 249
30	6.49	219, 250	Trapanin A (α or β)	1252–2	1176, 935, 785, 765, 613, 451, 313, 301, 275, 169, 125
31	6.62	219, 271	Tetra-*O*-galloyl-*β*-D-glucose	787	617, 483, 465, 331, 313, 169, 125
			IRIDOIDS		
32	3.88	238	Loganic acid	2M 751, 375	213, 169, 151
33	6.12	238	Loganin	389 (-) or 435 (+)	227, 209
34	8.05	238	Cornuside	2M 1083, 541	169, 125
			ANTHOCYANINS		
35	4.34	240, 515	Cyanidin 3-*O*-galactoside	449	287
36	4.51	242, 517	Cyanidin 3-*O*-robinobioside	595	287
37	4.71	271, 501	Pelargonidin 3-*O*-galactoside	433	271
38	4.99	271, 501	Pelargonidin 3-*O*-robinobioside	579	271
			FLAVONOLS		
39	6.86	352	Quercetin-3-*O*-glucuronide	477	301, 178, 151
			PHENOLIC ACIDS		
40	3.26	326	*trans*-caftaric acid isomer	311	179, 149
41	3.57	289	*p*-coumaric acid derivative	341	211, 195, 163
42	4.10	326	*cis*-caftaric acid isomer	311	179, 149
43	5.03	311	*trans*-coutaric acid	295	163, 149
44	5.07	310	*p*-coumaric acid	163	149
45	5.53	311	*cis*-coutaric acid	295	163, 149

Abbreviations: tR, retention time; MS1, the first mass spectrum (pseudomolecular ions); MS2, the second mass spectrum (fragment ions), HHDP, hexahydroxydiphenoyl. Main signals are underlined;

Table S3 HPLC-PDA quantification of the secondary metabolites and antioxidant capacity during the development and ripening of cornelian cherry fruits.

		PHENOLIC COMPOUNDS					IRIDOIDS	SECONDARY METABOLITES	ANTIOXIDANT CAPACITY	
		Phenolic acids [mg/100 g dw]	Hydrolysable tannins [mg/100 g dw]	Anthocyanins [mg/100 g dw]	Flavonols [mg/100 g dw]	TOTAL [mg/100 g dw]	Iridoids [mg/100 g dw]	TSM [mg/100 g dw]	ABTS [mM TE/100g dw]	FRAP [mM TE/100g dw]
Cultivar	Stage	*mean*	*mean*	*mean*	*mean*	*mean*	*mean*	*mean*	*mean ± SD*	*mean ± SD*
**'Bolestraszycki'**	S1	2587.36 c	1661.23 a		63.89 a	4312.47 b	2058.45 e	6370.92 b	102.12 ± 1.17 b	97.76 ± 0.85 a
	S2	1906.50 e	1532.19 b		61.79 a	3513.68 e	2109.34 e	5609.83 d	105.54 ± 2.74 b	71.61 ± 5.18 bc
	S3	1184.11 h	1054.42 d	0.00	42.25 b	2283.78 i	1717.21 gh	4000.99 h	74.11 ± 2.60 ef	61.33 ± 1.39 d
	S4	574.65 k	507.73 l		23.15 c	1105.54 lm	1238.16 jk	2343.69 k	64.14 ± 0.64 i	26.35 ± 0.16 g
	S5	572.02 k	340.78 no		15.11 ef	927.91 mn	1688.51 h	2616.42 lm	25.70 ± 0.83 kl	22.31 ± 1.92 gh
	S6	547.10 k	312.50 op	143.17 a	11.43 fg	1017.74 n	960.38 m	1974.59 n	9.78 ± 0.19 n	13.44 ± 0.35 i
**% P S1**		**60.0%**	**38.5%**	**0.0%**	**1.5%**	**100%**	**-**	**-**	**-**	**-**
**% P S6**		**53.8%**	**31.3%**	**14.1%**	**1.1%**	**100%**	**-**	**-**	**-**	**-**
**% TSM S1**		**40.6%**	**26.1%**	**0.0%**	**1.0%**	-	**32.3%**	**100%**	**-**	**-**
**% TSM S6**		**27.7%**	**15.8%**	**7.3%**	**0.6%**	-	**48.6%**	**100%**	**-**	**-**
**'Słowianin'**	S1	3561.64 a	1210.60 c		17.55 de	4789.78 a	1989.84 f	6779.62 a	76.31 ± 4.51 e	63.00 ± 4.57 d
	S2	3045.89 b	995.21 e		12.83 fg	4053.93 c	2282.50 d	6336.43 b	72.71 ± 0.40 efg	76.38 ± 0.21 b
	S3	2478.09 c	774.74 h	0.00	12.13 fg	3264.96 f	2112.15 e	5377.11 e	68.40 ± 2.03 hg	69.44 ± 1.24 c
	S4	1520.18 f	713.01 i		8.59 gh	2241.78 i	1692.42 h	3934.19 hi	69.23 ± 0.25 fg	46.36 ± 0.03 e
	S5	947.99 i	289.85 p		4.12 i	1241.96 kl	1291.87 j	2533.82 kl	23.78 ± 0.38 lm	20.35 ± 0.25 h
	S6	403.40 l	233.92 r	42.94 c	2.27 i	682.03 o	721.15 n	1403.67 p	20.21 ± 0.81 m	13.09 ± 1.47 i
**% P S1**		**74.4%**	**25.3%**	**0.0%**	**0.4%**	**100%**	**-**	**-**	**-**	**-**
**% P S6**		**59.1%**	**34.3%**	**6.2%**	**0.3%**	**100%**	**-**	**-**	**-**	**-**
**% TSM S1**		**52.5%**	**17.9%**	**0.0%**	**0.3%**	-	**29.4%**	**100%**	**-**	**-**
**% TSM S6**		**28.7%**	**16.7%**	**3.06%**	**0.2%**	-	**51.4%**	**100%**	**-**	**-**
**'Paczoski'**	S1	2185.29 d	948.20 f		21.85 cd	3155.34 f	2709.88 b	5865.22 c	92.94 ± 3.52 c	74.05 ± 5.53 bc
	S2	1877.67 e	887.40 g		19.61 cd	2784.69 h	3093.37 a	5878.05 c	110.73 ± 1.24 a	77.21 ± 5.04 b
	S3	1336.20 g	572.92 k	0.00	11.60 fg	1920.72 j	3108.56 a	5029.29 f	101.42 ± 1.85 b	59.62 ± 1.15 d
	S4	948.68 i	407.20 m		12.99 f	1368.87 k	2535.96 c	3904.82 hi	42.26 ± 0.30 j	35.37 ± 0.57 f
	S5	603.54 jk	146.29 pq		6.02 hi	893.02 n	2073.75 e	2829.60 j	29.45 ± 0.00 k	17.25 ± 0.13 hi
	S6	285.37 l	194.29 s	67.41 b	3.42 i	550.50 o	1126.19 l	1676.69 o	18.54 ± 0.26 n	5.32 ± 0.23 j
**% P S1**		**69.3%**	**30.1%**	**0.0%**	**0.7%**	**100%**	**-**	**-**	**-**	**-**
**% P S6**		**51.8%**	**35.3%**	**12.2%**	**0.6%**	**100%**	**-**	**-**	**-**	**-**
**% TSM S1**		**37.3%**	**16.2%**	**0.0%**	**0.4%**	-	**46.2%**	**100%**	**-**	**-**
**% TSM S6**		**17.0%**	**11.6%**	**4.0%**	**0.2%**	-	**67.2%**	**100%**	**-**	**-**
**'Florianka'**	S1	2937.17 b	869.49 g		23.46 c	3830.13 d	1758.94 gh	5589.07 d	76.54 ± 2.88 e	62.76 ± 0.10 d
	S2	2554.32 c	934.15 f		18.04 de	3506.51 e	1780.09 g	5286.60 e	86.51 ± 7.09 d	75.18 ± 3.23 bc
	S3	2187.18 d	782.35 h	0.00	19.96 cd	2989.50 g	1740.69 gh	4730.19 g	82.28 ± 0.26 d	70.44 ± 0.54 c
	S4	1556.10 f	651.32 j		18.34 de	2225.76 i	1535.28 i	3761.03 i	77.49 ± 0.01 e	58.38 ± 4.46 d
	S5	712.40 j	247.27 qr		12.51 fg	972.18 mn	1209.37 k	2181.55 m	63.73 ± 1.27 hi	35.71 ± 2.48 f
	S6	552.19 k	376.11 mn	30.83 d	6.41 hi	966.33 mn	910.17 m	1876.50 n	42.40 ± 2.07 j	31.73 ± 2.32 f
**% P S1**		**76.7%**	**22.7%**	**0.0%**	**0.6%**	**100%**	**-**	**-**	**-**	**-**
**% P S6**		**57.2%**	**38.9%**	**3.2%**	**0.7%**	**100%**	**-**	**-**	**-**	**-**
**% TSM S1**		**52.6%**	**15.6%**	**0.0%**	**0.4%**	-	**31.5%**	**100%**	**-**	**-**
**% TSM S6**		**29.5%**	**20.0%**	**1.6%**	**0.3%**	-	**48.5%**	**100%**	**-**	**-**

The results are expressed as mean (sums), and mean ± standard deviation (SD), n = 3. **Legend: TSM**, total secondary metabolites; S1-S6. subsequent sampling stages; % P S1, % P S6, percentage of individual group of compounds within polyphenols at the stages S1 and S6; % TSM S1, % TSM S6, percentage of individual group of compounds within total secondary metabolites at the stages S1 and S6;

Table S4 HPLC-PDA quantification [mg/100 g dw] of the individual polyphenols during the development and ripening of cornelian cherry fruit.

		**PHENOLIC ACIDS**						**ANTHOCYANINS**				**FLAVONOLS**
		***p*-coumaric acid deriv.**	**Caftaric acid I (*trans*)**	**Caftaric acid II (*cis*)**	**Coutaric acid I (*trans*)**	**Coumaric acid**	**Coutaric acid II (*cis*)**	**Cy-3-gal**	**Cy-3-rob**	**Pg-3-gal**	**Pg-3-rob**	**Q-3-glucur**
Cultivar		*mean ± SD*	*mean ± SD*	*mean ± SD*	*mean ± SD*	*mean ± SD*	*mean ± SD*	*mean ± SD*	*mean ± SD*	*mean ± SD*	*mean ± SD*	*mean ± SD*
'Bolestraszycki'	**S1**	6.18 ± 0.15 a	524.58 ± 12.34 a	974.51 ± 1.60 f	120.36 ± 1.91 d	4.09 ± 0.01 ab	957.63 ± 5.32 a					63.89 ± 3.27 a
	**S2**	7.01 ± 0.30 b	422.42 ± 15.59 c	740.24 ± 20.93 h	97.57 ± 4.46 e	3.14 ± 0.01 de	636.13 ± 5.55 d					61.79 ± 6.02 a
	**S3**	5.64 ± 0.06 c	289.06 ± 2.19 f	452.24 ± 10.23 i	71.32 ± 0.36 g	2.12 ± 0.19 ghi	363.73 ± 0.20 j	0.00	0.00	0.00	0.00	42.25 ± 0.41 b
	**S4**	3.33 ± 0.04 hi	148.63 ± 3.67 hi	216.08 ± 6.41 k	32.16 ± 2.01 k	1.55 ± 0.03jklm	172.91 ± 3.25 o					23.15 ± 0.69 c
	**S5**	2.97 ± 0.23 ijk	134.46 ± 8.04 i	215.63 ± 12.99 k	22.74 ± 1.66 klm	1.96 ± 0.28 hijk	194.26 ± 11.55 n					15.11 ± 0.37 ef
	**S6**	3.15 ± 0.08 ij	201.19 ± 2.54 g	276.49 ± 4.14 jk	12.80 ± 0.08 no	0.35 ± 0.03 o	53.12 ± 0.03 r	15.39 ± 0.17 b	18.40 ± 0.20 a	74.29 ± 0.82 a	35.10 ± 0.39 a	11.43 ± 0.11 fg
% S1		**0.2%**	**20.3%**	**37.7%**	**4.7%**	**0.2%**	**37%**	**-**	**-**	**-**	**-**	**100%**
% S6		**0.6%**	**36.8%**	**50.5%**	**2.3%**	**0.1%**	**9.7%**	**10.7%**	**12.9%**	**51.9%**	**24.5%**	**100%**
'Słowianin'	**S1**	4.22 ± 0.08 ef	457.31 ± 6.09 b	2431.67 ± 61.49 a	61.22 ± 1.29 h	3.45 ± 0.11 cd	603.77 ± 18.53 e					17.55 ± 1.26 ed
	**S2**	3.63 ± 0.26 gh	402.34 ± 6.44 cd	2081.32 ± 65.05 b	52.27 ± 1.14 i	2.77 ± 0.81 ef	503.57 ± 4.39 g					12.83 ± 0.50 fg
	**S3**	4.63 ± 0.07 d	336.97 ± 2.33 e	1700.36 ± 1.08 d	41.75 ± 0.31 j	2.54 ± 0.05 fg	391.82 ± 1.50 i	0.00	0.00	0.00	0.00	12.13 ± 0.47 fg
	**S4**	3.95 ± 0.23 fg	221.23 ± 22.05 g	1034.34 ± 33.45 f	30.82 ± 0.98 k	1.72 ± 0.14 ijk	228.11 ± 1.50 m					8.59 ± 0.09 gh
	**S5**	3.33 ± 0.33 hi	128.57 ± 5.90 i	667.59 ± 55.53 h	16.91 ± 2.04 mno	1.45 ± 0.13 klm	130.14 ± 15.26 p					4.12 ± 0.17 i
	**S6**	2.92 ± 0.01 jk	60.91 ± 1.34 j	278.69 ± 22.78 jk	11.11 ± 0.59 o	0.45 ± 0.03 o	49.32 ± 2.28 r	15.99 ± 0.33 b	1.91 ± 0.08 b	23.27 ± 1.17 c	1.76 ± 0.06 c	2.27 ± 0.04 i
% S1		**0.1%**	**12.8%**	**68.3%**	**1.7%**	**0.1%**	**17.0%**	**-**	**-**	**-**	**-**	**100%**
% S6		**0.7%**	**15.1%**	**69.1%**	**2.8%**	**0.1%**	**12.2%**	**37.2%**	**4.4%**	**54.2%**	**4.1%**	**100%**
'Paczoski'	**S1**	4.50 ± 0.26 de	324.82 ± 13.82 e	852.09 ± 7.35 g	81.52 ± 2.36 f	4.47 ± 0.54 a	917.89 ± 1.66 b					21.85 ± 0.77 c
	**S2**	4.77 ± 0.46 d	297.23 ± 23.37 f	740.06 ± 6.10 h	61.85 ± 0.67 h	3.81 ± 0.17 ab	769.96 ± 16.70 c					19.61 ± 2.78 ed
	**S3**	5.72 ± 0.01 c	219.73 ± 0.86 g	487.50 ± 0.85 i	41.21 ± 0.17 j	3.34 ± 0.23 cd	578.69 ± 8.45 f	0.00	0.00	0.00	0.00	11.60 ± 0.71 fg
	**S4**	6.01 ± 0.14 bc	169.51 ± 6.57 h	357.49 ± 15.76 j	29.42 ± 0.14 kl	2.38 ± 0.32 fgh	383.87 ± 4.03 i					12.99 ± 0.22 f
	**S5**	3.86 ± 0.02 fg	127.94 ± 1.07 i	217.07 ± 2.42 k	21.26 ± 0.05 lmn	2.32 ± 0.06 fgh	231.10 ± 4.40 m					6.02 ± 0.16 fg
	**S6**	2.71 ± 0.04 kl	69.13 ± 12.74 j	102.72 ± 10.25 l	15.91 ± 1.20 mno	1.10 ± 0.23 mn	93.81 ± 2.89 q	20.34 ± 2.47 a	0.54 ± 0.04 d	45.18 ± 4.68 b	1.36 ± 0.19 d	3.42 ± 0.27 i
% S1		**0.2%**	**14.9%**	**39.0%**	**3.7%**	**0.2%**	**42.0%**	**-**	**-**	**-**	**-**	**100%**
% S6		**0.9%**	**24.2%**	**36.0%**	**5.6%**	**0.4%**	**32.9%**	**30.2%**	**0.8%**	**67.0%**	**2.0%**	**100%**
'Florianka'	**S1**	1.25 ± 0.01 n	398.31 ± 15.65 cd	1929.26 ± 92.40 c	183.52 ± 1.29 b	2.04 ± 0.25 ghij	422.79 ± 13.21 h					23.46 ± 1.38 c
	**S2**	1.59 ± 0.07 mn	385.38 ± 12.31 d	1654.55 ± 69.67 d	185.08 ± 14.76 b	1.69 ± 0.23 ijkl	326.03 ± 8.75 k	0.00	0.00	0.00	0.00	18.04 ± 3.05 ed
	**S3**	2.49 ± 0.11 l	405.53 ± 7.95 cd	1286.61 ± 3.11 e	205.23 ± 1.58 a	1.39 ± 0.01 klm	285.93 ± 3.11 l					19.96 ± 0.02 cd

	**S4**	2.35 ± 0.04 l	335.13 ± 21.87 e	877.43 ± 88.08 g	157.15 ± 10.47 c	1.14 ± 0.14 lmn	182.91 ± 9.50 no					18.34 ± 3.28 ed
	**S5**	1.70 ± 0.08 m	123.20 ± 29.30 i	308.66 ± 60.86 jk	148.80 ± 3.77 c	0.70 ± 0.07 no	129.33 ± 2.87 p					12.51 ± 0.29 fg
	**S6**	0.78 ± 0.03 o	122.80 ± 7.48 i	294.90 ± 14.39 jk	87.17 ± 6.31 f	0.33 ± 0.05 o	47.00 ± 2.15 r	3.56 ± 0.08 c	1.58 ± 0.16 c	22.03 ± 0.36 c	3.67 ± 0.62 b	6.41 ± 0.90 hi
% S1		**0.0%**	**13.6%**	**65.7%**	**6.2%**	**0.1%**	**14.4%**	**-**	**-**	**-**	**-**	**100%**
% S6		**0.1%**	**22.2%**	**53.3%**	**15.8%**	**0.1%**	**8.5%**	**11.5%**	**5.1%**	**71.4%**	**11.9%**	**100%**

The results are expressed as mean ± standard deviation (SD), n = 3. Abbreviations: **S1-S6**. subsequent sampling stages; **% share S1**, **% share S6**, percentage of individual compound within a group at the stages S1 and S6, respectively; **dw**. dry weight; small letters stand as homogenous groups (Duncan's test); **Cy 3-gal**. cyanidin 3-*O*-galactoside; **Cy-3-rob**. cyanidin 3-*O*-robinobioside; **Pg-3-gal**. pelargonidin 3-*O*-galactoside; **Pg-3-rob**. pelargonidin 3-*O*-robinobioside; **Q-3-glucur**. quercetin 3-*O*-glucuronide;

Table S5 HPLC-PDA quantification [mg/100 g dw] of the individual hydrolysable tannins and iridoids during the development and ripening of cornelian cherry fruit.

		HYDROLYZABLE TANNINS							IRIDOIDS	
		**Gallic acid**	**Ellagic acid**	**Gallotannins**	**Camptothin A isomers**	**Cornusiin A isomers**	**Cornusiin F isomers**	**Cornusiin C isomers**	**Loganic acid**	**Cornuside**
Cultivar	Stage	*mean ± SD*	*mean ± SD*	*mean ± SD*	*mean ± SD*	*mean ± SD*	*mean ± SD*	*mean ± SD*	*mean ± SD*	*mean ± SD*
'Bolestraszycki'	S1	69.73 ± 0.90 c	3.51 ± 0.31 hi	241.05 ± 3.44 c	477.55 ± 5.77 a	350.75 ± 3.18 a	212.70 ± 5.47 a	225.34 ± 2.21 a	1700.96 ± 12.06 h	357.48 ± 12.39 f
	S2	48.71 ± 0.49 de	6.20 ± 0.43 ef	300.81 ± 16.62 b	384.88 ± 11.81 b	328.11 ± 11.91 b	201.20 ± 1.38 b	180.04 ± 1.00 b	1817.14 ± 4.75 f	292.20 ± 2.67 g
	S3	42.91 ± 1.76 efg	3.85 ± 0.09 h	331.08 ± 2.19 a	199.92 ± 2.02 g	200.50 ± 13.12 f	99.64 ± 9.74 f	83.30 ± 4.81 g	1567.97 ± 28.50 i	149.24 ± 2.02 k
	S4	36.43 ± 1.18 gh	1.28 ± 0.03 kl	225.07 ± 11.19 d	93.21 ± 3.34 jk	64.76 ± 7.39 k	28.45 ± 2.69 ij	45.01 ± 2.66 lm	1153.62 ± 3.13 m	84.54 ± 4.64 m
	S5	25.12 ± 3.57 j	1.32 ± 0.01 kl	72.69 ± 5.85 m	89.56 ± 0.66 k	70.12 ± 0.08 k	25.87 ± 0.90 ijkl	42.14 ± 2.64 lm	1572.70 ± 61.05 i	115.81 ± 9.92 l
	S6	5.80 ± 0.30 kl	2.10 ± 0.66 jk	143.68 ± 1.64 g	54.13 ± 0.79 lm	24.05 ± 0.85 o	26.47 ± 0.91 ijk	38.45 ± 0.76 mn	900.83 ± 6.08 o	59.55 ± 0.06 n
% S1		**4.2%**	**0.2%**	**14.5%**	**28.7%**	**21.1%**	**12.8%**	**13.6%**	**82.6%**	**17.4%**
% S6		**1.8%**	**0.7%**	**50.0%**	**17.3%**	**7.7%**	**8.5%**	**12.3%**	**93.1%**	**6.9%**
'Słowianin'	S1	49.19 ± 1.58 de	1.16 ± 0.28 kl	125.99 ± 0.85 hi	334.78 ± 1.58 c	287.70 ± 2.62 c	147.51 ± 3.60 c	142.34 ± 3.05 c	1508.15 ± 51.90 j	481.69 ± 6.72 c
	S2	48.97 ± 0.96 de	6.22 ± 0.73 f	87.27 ± 3.88 lm	299.36 ± 0.34 e	240.09 ± 4.08 d	112.47 ± 4.60 e	113.91 ± 2.09 e	1778.42 ± 38.62 fg	504.08 ± 16.72 b
	S3	39.03 ± 0.85 fgh	6.04 ± 0.47 f	139.82 ± 14.17 gh	210.53 ± 4.44 g	156.97 ± 2.50 g	81.96 ± 0.56 g	72.83 ± 5.52 h	1728.25 ± 7.52 gh	383.91 ± 4.56 e
	S4	28.41 ± 0.11 ij	4.77 ± 0.08 g	242.62 ± 5.34 c	139.75 ± 5.46 i	123.76 ± 1.33 i	52.22 ± 2.27 h	53.77 ± 3.10 j	1456.06 ± 12.39 j	236.36 ± 1.62 i
	S5	26.12 ± 6.43 j	2.06 ± 0.43 jk	101.98 ± 12.38 jkl	62.78 ± 4.82 l	51.88 ± 8.37 l	19.89 ± 1.22 klm	22.79 ± 5.26 p	1148.16 ± 65.56 m	143.70 ± 14.99 k
	S6	0.00 ± 0.00 l	0.94 ± 0.01 l	106.84 ± 1.99 jk	44.40 ± 2.93 n	26.64 ± 2.82 mn	15.50 ± 0.43 m	22.88 ± 2.33 p	661.74 ± 4.70 q	59.41 ± 0.36 n
% S1		**4.1%**	**0.1%**	**10.4%**	**27.7%**	**23.8%**	**12.2%**	**11.8%**	**75.8%**	**24.2%**
% S6		**0.0%**	**0.4%**	**45.7%**	**19.0%**	**11.4%**	**6.6%**	**9.8%**	**91.8%**	**8.2%**
'Paczoski'	S1	37.60 ± 4.60 gh	4.24 ± 1.04 gh	20.43 ± 0.04 p	292.58 ± 0.19 e	250.17 ± 1.29 d	128.89 ± 0.40 d	132.72 ± 0.24 d	2295.61 ± 26.31 d	414.27 ± 5.45 d
	S2	44.38 ± 0.48 ef	5.85 ± 0.26 e	37.95 ± 0.95 no	256.04 ± 1.16 f	226.99 ± 1.03 e	128.06 ± 2.09 d	115.85 ± 4.12 e	2708.85 ± 3.72 b	384.52 ± 0.36 e
	S3	33.77 ± 5.67 hi	2.85 ± 0.04 ij	47.76 ± 0.43 n	159.77 ± 1.81 h	136.37 ± 1.86 h	45.66 ± 1.15 h	64.42 ± 3.85 i	2839.38 ± 4.22 a	269.18 ± 1.81 h
	S4	27.57 ± 0.01 ij	1.74 ± 0.01 kl	42.26 ± 0.46 no	105.88 ± 0.07 j	103.41 ± 1.03 j	29.86 ± 0.59 i	46.52 ± 0.36 kl	2360.53 ± 0.61 c	175.43 ± 1.66 j
	S5	29.92 ± 0.28 ij	1.63 ± 0.12 kl	28.21 ± 1.43 op	67.16 ± 1.77 l	68.99 ± 4.05 k	21.76 ± 0.00 jklm	32.68 ± 3.08 no	1943.45 ± 4.56 e	130.30 ± 0.35 kl
	S6	3.53 ± 0.54 l	1.99 ± 0.38 jk	27.91 ± 1.49 op	48.39 ± 7.06 n	23.60 ± 3.85 o	14.87 ± 3.82 m	25.68 ± 0.28 p	1056.73 ± 0.23 n	69.46 ± 15.26 mn
% S1		**4.0%**	**0.5%**	**2.1%**	**30.9%**	**26.4%**	**13.6%**	**14.0%**	**84.7%**	**15.3%**
% S6		**0.0%**	**1.0%**	**14.4%**	**24.9%**	**12.1%**	**7.7%**	**13.2%**	**93.8%**	**6.2%**
'Florianka'	S1	91.39 ± 0.91 a	9.33 ± 0.47 d	113.21 ± 2.97 ij	253.39 ± 15.86 d	143.02 ± 6.90 h	116.24 ± 0.95 e	59.05 ± 3.82 ij	1206.69 ± 12.31 m	552.25 ± 12.31 a

	S2	84.45 ± 5.32 b	9.26 ± 0.43 d	194.64 ± 16.30 e	258.98 ± 15.19 f	158.81 ± 3.30 g	113.59 ± 0.40 e	91.50 ± 1.46 f	1307.26 ± 30.43 l	472.83 ± 15.77 c
	S3	80.48 ± 5.14 b	31.62 ± 0.55 a	289.34 ± 5.94 b	166.46 ± 2.85 h	75.00 ± 8.67 k	95.17 ± 2.27 f	53.01 ± 5.15 jk	1392.98 ± 9.17 k	347.71 ± 2.76 f
	S4	54.28 ± 2.80 d	12.73 ± 0.11 c	290.19 ± 6.97 b	97.39 ± 3.87 jk	76.11 ± 8.53 k	52.28 ± 7.33 h	67.44 ± 3.73 i	1305.98 ± 12.43 l	229.30 ± 18.51 i
	S5	45.71 ± 4.80 e	13.57 ± 0.29 b	92.67 ± 11.91 kl	22.06 ± 4.22 o	31.79 ± 6.18 mn	7.58 ± 1.24 n	29.59 ± 3.54 op	1151.50 ± 31.09 m	57.87 ± 9.49 n
	S6	10.32 ± 0.54 k	2.76 ± 0.04 ij	162.82 ± 7.83 f	66.77 ± 2.16 l	37.42 ± 0.03 m	18.89 ± 0.89 lm	46.63 ± 2.04 kl	835.03 ± 19.74 p	75.14 ± 0.92 mn
% S1		**10.5%**	**1.1%**	**13.0%**	**29.1%**	**16.4%**	**13.4%**	**6.8%**	**68.6%**	**31.4%**
% S6		**2.7%**	**0.7%**	**43.3%**	**17.8%**	**9.9%**	**5.0%**	**12.4%**	**91.7%**	**8.3%**

The results are expressed as mean ± standard deviation (SD), n = 3. Abbreviations: **S1-S6**, subsequent sampling stages; **% S1**, **% S6**, percentage of individual compound within a group at the stages S1 and S6, respectively; **dw**, dry weight; small letters stand as homogenous groups (Duncan’s test);

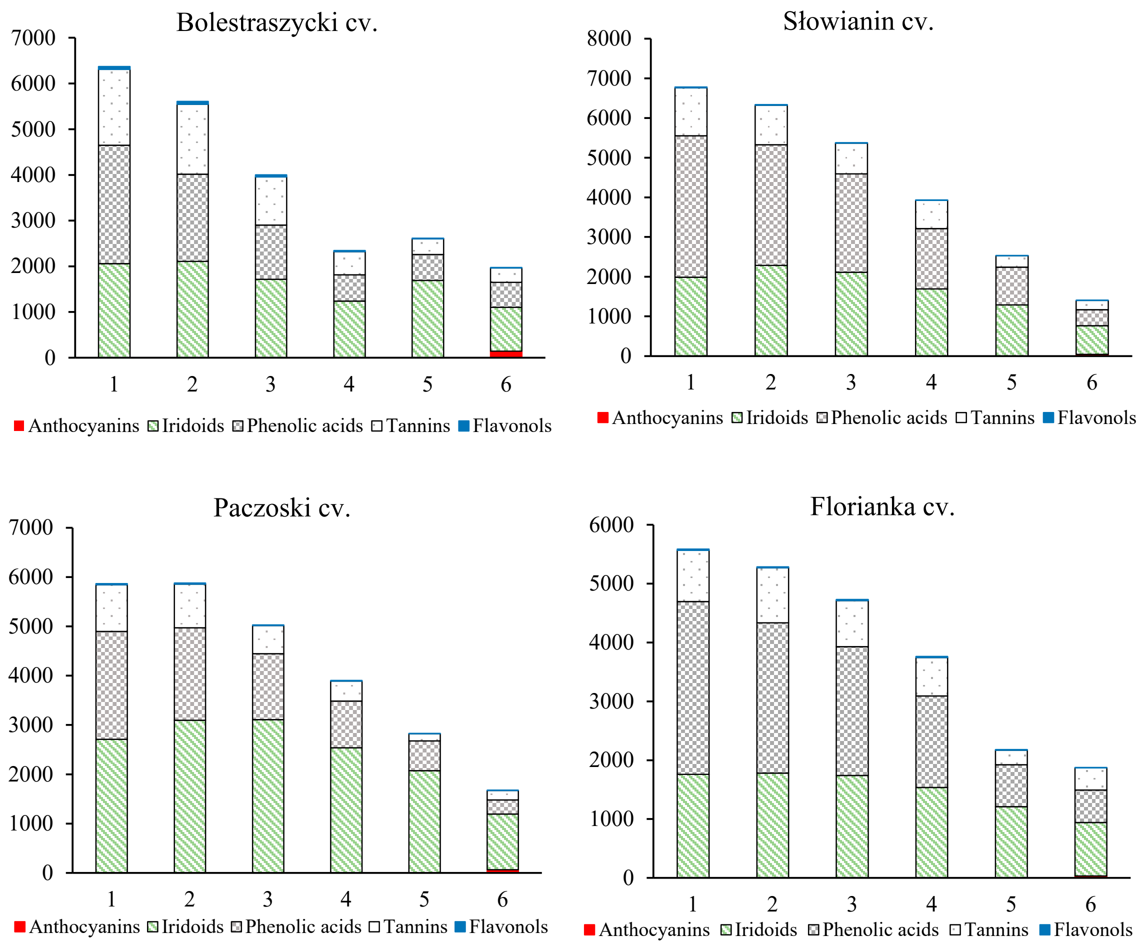

Figure S1 Changes in the content (mg/100 g dw) all groups of analyzed secondary metabolites during the development and ripening of cornelian cherry. The numbers (1–6) on the horizontal axis represent studied stages. Small letters stand as homogenous groups (Duncan's test).
